# Role of Human Papilloma Virus 16/18 In Laryngeal Carcinoma with Correlation to the Expression of Cyclin D1, p53, p16 and EGFR

**DOI:** 10.12669/pjms.39.6.8220

**Published:** 2023

**Authors:** Naila Tariq, Talat Mirza, Maqsood Ansari, Sobia Nazir

**Affiliations:** 1 Naila Tariq HOD, Laboratory National Medical Center, Karachi, Pakistan; 2 Talat Mirza Meritorious Professor of Pathology Department of Research & Molecular Medicine Ziauddin University, Karachi, Pakistan; 3 Maqsood Ali Ansari. Professor & HOD, Department of Genetics, University of Karachi, Karachi, Pakistan; 4 Sobia Nazir, MPhil. Molecular Pathology Reliance Laboratory, Karachi, Pakistan

**Keywords:** Laryngeal Squamous Cell Carcinoma, Human Papilloma Virus 16, Human Papilloma Virus 18, Tumor suppressor genes, Polymerase chain reaction

## Abstract

**Objective:**

To investigate the role of Human Papilloma Virus (HPV) (16/18) in relation to the molecular genetic mechanisms of Cyclin D1, p53, p16, and Epidermal growth factor receptor (EGFR) in Laryngeal Squamous Cell Carcinoma (LSSC)

**Methods:**

A cross-sectional study of 88 (Formalin-fixed Paraffin Embedded) FFPE laryngeal biopsies were done at Basic Medical Sciences Institute, Jinnah Postgraduate Centre, Karachi from 2010 to 2019 with the application of Polymerase chain reaction (PCR) for HPV 16/18and Immuno-histochemical staining for molecular genetic expression of proteins, Cyclin D1, p53, p16, and EGFR.

**Results:**

Out of 88 cases of Laryngeal Squamous Cell Carcinoma (LSSC) there was female preponderance. Mean age of the participants was found as 50.7±12.8 years. High risk HPV 16/18 was positive in 28 cases (31.8%), largely related to Grade-II and Grade-III. Immunohistochemically, Cyclin D1 (87.5%) appeared as the most important driver mutation followed by p16 (86.4%), EGFR (65.9%), and, p53 was positive in (61.4%) of cases.

**Conclusion:**

The role of high-risk HPV 16/18 is concurred in the present study strongly in correlation to p16 as a surrogate marker. Moreover, the other driver mutations of Cyclin D1, p53, and EGFR are also implicated as cumulative molecular events in tumor progression as mostly seen in higher Grades.

## INTRODUCTION

Head and neck cancers are one of the most prevalent cancers across the world, Laryngeal Squamous Cell Carcinoma (LSSC) being the 21^st^ most frequent malignancy. The cancer burden is expected to be 28.4 million cases by 2040^1^, 47% rise from 2020 with larger increase in transitioning versus transitional countries due to demographic changes, risk factors along with associated globalization. According to the American Cancer Society, the most recent estimate from 2020, showed approximately 54010 new cases of head and neck carcinomas in United States, of these 37260 were in men and 16750 were seen in women.^2^ The overall survival rate being 65%.

Cumulative molecular events lead to progressive transformation in relation to oncogenesis. Cyclin D is a proliferative oncogene which plays a central role in the regulation of proliferation linking the extracellular signaling environment to cell cycle progression. CD1 over expression helps the earlier identification of risk of head and neck cancer. Over expression in LSCC is implicated with biological behavior of the tumor with valuable prognostic significance.^3^ p53 is a tumor suppressor gene, activated p53 promotes the cell cycle arrest to allow DNA repair or apoptosis preventing propagation of cells with DNA damage. Mutation of p53 leads to cancer progression. Over expression of p53 is associated with better survival of cancer.^4^ Cyclin D1 and p53 are considered as prognostic markers of tumor proliferation in laryngeal carcinoma. p16 protein is used as a surrogate marker for identifying HPV infection. p16 is a tumor suppressor protein. EGFR influences tumor growth and the neoplastic transformation. High expression of EGFR is connected to aggressive tumor behavior and high risk metastasis and treatment failure.

The risk factors related to laryngeal carcinoma includes genetic predisposition, tobacco, alcohol, prolonged exposure to work place chemicals, Gastro esophageal reflux disease (GERD), poor nutrition, HPV infection^5^ and exposure to asbestos and Nickle. Mutation of certain genes that can lead to development of laryngeal cancer include one of the most commonly implicated p53. Other genes that may be mutated in laryngeal carcinoma are, CDKN2A, PTEN and EGFR.^6^,^7^ Structural Chromosomal abnormalities such as deletions, translocation and amplification, can affect the expression or function of critical cell cycle regulators, DNA repair and apoptotic genes. Epigenetic modifications which involve gene expression without changes in the underlying DNA sequence have been implicated in laryngeal carcinoma like DNA methylation, histone modifications and micro-RNA dysregulation.

Moreover, dysregulation of signaling pathways like the P13k/AKT/mToR pathways are also further implicated. Although the role of HPV virus in laryngeal carcinoma is still not clear, however, prevalence of HPV in laryngeal carcinoma has been proven in different studies varying from 2.4% to 76.42%.^8^ There are studies showing role of HPV in cancer progression and its association with increased accessibility and proliferation of basal layers. Human papilloma viruses dysregulate the cell cycle and leads to apoptosis. The viral replication produces proteins E1 & E2 playing a role in the amplification of the viral genome whereas E6 and E7 proteins are complexed with tumor suppressors p53 and pRb leading to anti-apoptosis & genetic instability.^9^

The LSSC is on the rise in our genetically distinct population which needs to be investigated for the molecular pathogenesis to assess the local mutation landscape. Therefore, we investigated the role of HPV infection in laryngeal carcinoma in relation to the molecular genetic mechanisms of Cyclin D1, p53, p16 and EGFR.

## METHODS

A cross-sectional study was done in collaboration with the Histopathology Department of the Basic Medical Sciences Institute and the Clinical Pathology Department at Jinnah Postgraduate Medical Centre, Karachi. A total of 88 cases of LSSC were submitted for histological diagnosis from 2010 to 2019. This was a prospective study which used FFPE blocks for immunohistochemical staining and PCR evaluation. All biopsy proven cases of LSSC were included, and the tissue blocks with poor fixation or inadequate tissue material were excluded. The proforma included demographic details like age, gender and grades of LSSC with details of immunohistochemistry markers and HPV status were assessed.

### Ethical Approval:

Ethical approval was taken from the Ethical Board of JPMC Ref No# F.2-81/2022 Geni / 305/JPMC dated 08-10-2022.

Routine processing and staining by Hematoxylin & Eosin was followed by histological diagnosis and grading of the tumor using Anneroth’s grading system.^10^ Sections from FFPE blocks were subjected to immunohistochemical staining of p53 (Thermoscientific), p16 (Roche, VENTANA PD-L1 SP263 Monoclonal Antibody), Cyclin D1 (Thermoscientific), and EGFR (Dako, Clone H11 Kodenr).

**Fig.1:**
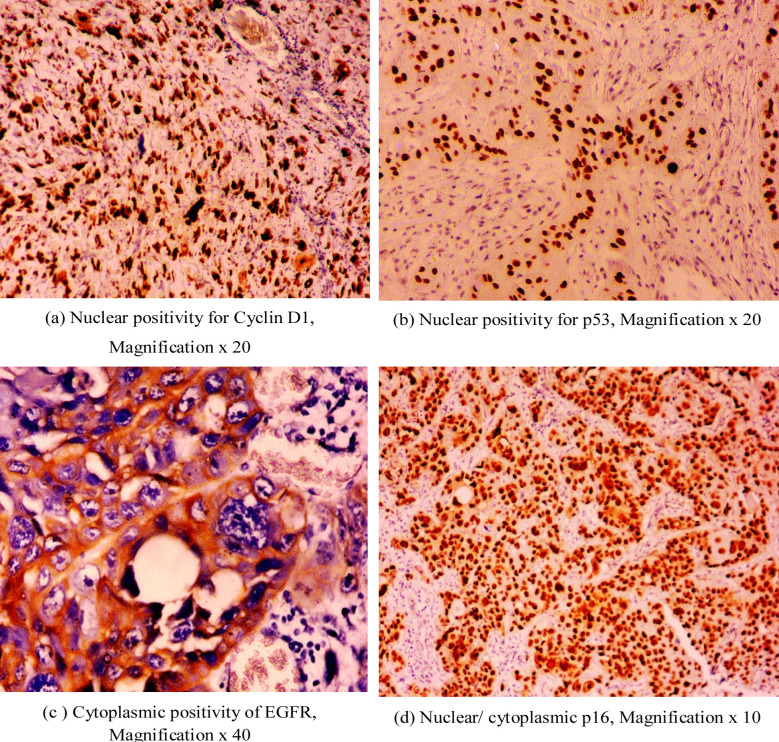
Photomicrographs.

The sections were routinely processed for dehydration and preparation of tissue for treatment of antigen retrieval. This was followed by incubation with the primary antibody which recognizes the target protein of interest. Tissue sections were then incubated with the secondary antibody conjugated to immunoperoxidase enzyme. The sections were treated with substrates making a visible signal for the localization of the target protein. Finally, the tissue was counter stained with hematoxylin to enhance the contrast and visualization of tissue morphology. Scoring for the immune expression was subsequently done as per semi quantitative scoring system based on reaction intensity and the number of labelled cells which are then calculated as cumulative expression score.^11^ Immunohistochemical Staining Localization were as follows: Cyclin D1 (nuclear), p53(nuclear), EGFR(cytoplasmic), p16(cytoplasmic + nuclear) (Fig.1).

### PCR:

The viral DNA was extracted by using the QIAamp DNA minikit in 1.5 ml microcentrifuge tube. In order to evaluate the quality of extracted DNA from paraffin embedded tissues and identify the potential inhibitions, PCR analysis was conducted using PCO3 & PCO4 primers that are specific for housekeeping genes (β-globin gene)^12^ Once the DNA quality was verified, HPV detection and genotyping were carried out using four sets of specifically designed primers. The first set consisted of GP5/GP6, which served as general primers for targeting HPV.

The remaining two sets of primers, namely TS16-A/TS16-B and Ts18-A/TS18-B, were subtype-specific primers designed for detecting subtype 16 and 18 of HPV, respectively. The amplification procedure was carried as a series of heating and cooling events to denature the DNA and the primers to extend the target DNA using Taq polymerase. During the reaction, the florescence of the probe was plotted as a graph of florescence signal versus cycle number, consequently the cycle threshold (Ct) was determined. PCR amplification and detection of target gene (L1) of HPV from FFPE blocks was done where the thermal profile used for PCR amplification was single cycle at 95°C for 5 minutes, 40 cycles at 94°C for 30 seconds, 45°C for 30 seconds, 72°C for 30 seconds and final extension of 5 minutes performed at 72°C. The annealing and melting temperatures for HPV subtype 16 and 18 were 61°C and 63°C respectively.

**Table:** Primers used for β-globin Gene, HPV (General Primers) L1.

Target gene	Primer name	Sequence of primer	Amplicon length
β-Globin	PCO3	ACACAACTGTGTTCACTAGC	110 bp
	PCO4	CAACTTCATCCACGTTCACC	
L1	GP5	TTTGTTACTGTGGTAGATAC	155 bp
	GP6	GAAAAATAAACTGTAAATCA

Primers used for HPV genotype 16 & 18			

*Target gene*	*Primer name*	*Sequence of primer*	*Amplicon length*

HPV 16	TS16-A	GGTCGGTGGACCGGTCGATG	96 bp
	TSI6-B	GCAATGTAGGTGTATCTCCA	
HPV 18	TS18-A	CCTTGGACGTAAATTTTTGG	115 bp
	TS18-B	CACGCACACGCTTGGCAGGT

For validation of our data, DNA extracted from cervical carcinomas which was identified to be positive for HPV subtype 16/18 were used as positive controls in PCR runs whereas negative control was PCR run without DNA template.

All the statistical analysis was done by using SPSS Version 24. Age and IHC expression scores were represented by Mean ± SD while age groups, gender, grades of Squamous Cell Carcinoma, HPV status (positive/negative) and IHC Markers’ characteristics were expressed in terms of frequency and percentages. The IHC expression scores were compared with grades of Squamous Cell Carcinoma by using ANOVA. The association of IHC markers with HPV status was assessed by applying Chi-square/Fisher’s exact test (for frequency ≤5 in any cell). P-value ≤ 0.05 was considered statistically significant.


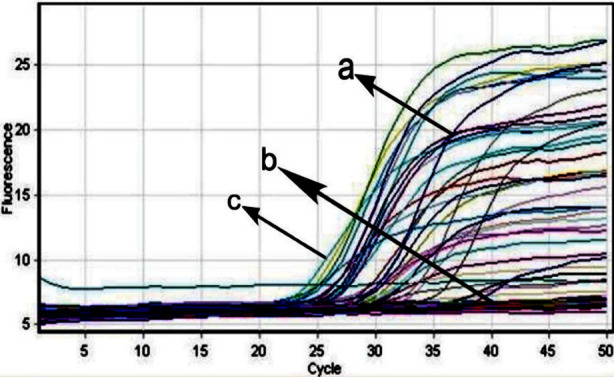


Real time PCR data of HPV General Primers: **(a)** Positive Sample for HPV **(b)** Negative sample for HPV and **(c)** Positive control for HPV.

## RESULTS

Our study showed a slight female preponderance. Mean age of the participants was found as 50.7±12.8 years. In the present study there were 88 samples in which 10 (27.8%) HPV positive cases were found having age up to 45 years and 18 (34.6%) HPV positive cases were found having age more than 45 years. Overall HPV positive status was found in 19 (41.3%) females and nine (21.4%) males, corresponding to the grades as one (33.3%) in Grade-I, seven (31.8%) in Grade-II, 15 (31.9%) in Grade-III and 5(31.8%) in Grade-IV, (Table-I).

**Table-I:** Stratification of HPV status with respect to Age group, Gender &Grades of Squamous cell carcinoma.

Variables		HPV status		Total	Chi-square value	P-value

		Positive	Negative			
Age groups	≤ 45 years	10 (27.8%)	26 (72.2%)	36 (100%)	0.458	0.498
	>45 years	18 (34.6%)	34 (65.4%)	52 (100%)		
	Total	28 (31.8%)	60 (68.2%)	88 (100%)		
Gender	Male	9 (21.4%)	33 (78.6%)	42 (100%)	3.998	0.046*
	Female	19 (41.3%)	27 (58.7%)	46 (100%)		
	Total	28 (31.8%)	60 (68.2%)	88 (100%)		
Grades of Squamous Cell Carcinoma	I	1 (33.3%)	2 (66.7%)	3 (100%)	0.282	>0.999
	II	7 (31.8%)	15 (68.2%)	22 (100%)		
	III	15 (31.9%)	32 (68.1%)	47 (100%)		
	IV	5 (31.2%)	11 (68.8%)	16 (100%)		
	Total	28 (31.8%)	60 (68.2%)	88 (100%)		

* p≤0.05 considered statistically significant results.

Out of the total 88 confirmed cases of laryngeal squamous cell carcinoma, HPV evaluation showed 60 cases negative (68.2%) while 28 cases were positive (31.8%). Out of 28 HPV positive cases 11 were noted other than 16/18 (not categorized), while in Type-16 there were 14 cases with four males and ten females and in Type-18 there were three cases found only in females.

In Table-II, Immuno-histochemical marker Cyclin D1 expressed as 87.5% followed by p16 with 86.4% positive cases. EGFR was positive in 65.9% cases whereas p53 positivity was seen in 61.4%. It also represents the comparison of Immunohistochemical expression scores with grades of Squamous Cell Carcinoma which displays highly significant correlation of all the markers with increasing grades having P-value ≤ 0.05 for each IHC marker.

**Table-II:** Comparison of Immunohistochemical Expression Scores with Grades.

Expression Scores	N	Grade-I	Grade-II	Grade-III	Grade-IV	F-statistic	p-value

		Mean (±SD)	Mean (±SD)	Mean (±SD)	Mean (±SD)		
Cyclin D1	77	4 (±0)	7.89 (±3.03)	7.6 (±3.2)	11 (±1.46)	5.998	<0.01*
p53	54	Nil	6.33 (±1.91)	9.5 (±3.02)	11.1 (±1.4)	13.309	<0.01*
p16	76	Nil	2 (±1.15)	6.27 (±2.83)	10.5 (±3)	10.398	<0.01*
EGFR	58	7 (±1.73)	9.06 (±3.36)	8.52 (±3.46)	11.4 (±1.81)	2.823	0.04*

* p<0.05 was considered statistically significant using One Way ANOVA.

The relationship of IHC markers with HPV status. p16 is highly positive especially in relation to HPV status (P-value = 0.008). Table-III. EGFR is also showing significant correlation with HPV positivity indicating an augmented role (P-value = 0.032). Hence, all HPV positive cases were noted to show positive staining for p16 as a surrogate marker.

**Table-III:** Correlation of Immune histochemical markers with HPV.

IHC Markers	HPV		Total	Chi-square value	P-value

	Positive	Negative			
CYCLIN D1 – Positive	23 (29.9%)	54 (70.1%)	77 (100%)	__	0.316
Negative	5 (45.5%)	6 (54.5%)	11 (100%)		
Total	28 (31.8%)	60 (68.2%)	88 (100%)		
p53 - Positive	17 (31.5%)	37 (68.5%)	54 (100%)	0.007	0.932
Negative	11 (32.4%)	23 (67.6%)	34 (100%)		
Total	28 (31.8%)	60 (68.2%)	88 (100%)		
p16 - Positive	28 (36.8%)	48 (63.2%)	76 (100%)	__	0.008*
Negative	0 (0%)	12 (100%)	12 (100%)		
Total	28 (31.8%)	60 (68.2%)	88 (100%)		
EGFR - Positive	23 (39.7%)	35 (60.3%)	58 (100%)	__	0.032*
Negative	5 (16.7%)	25 (83.3%)	30 (100%)		
Total	28 (31.8%)	60 (68.2%)	88 (100%)		

* p≤0.05 considered statistically significant results.

## DISCUSSION

Laryngeal squamous cell carcinoma is well established model for the cumulative molecular events in relation to viral carcinogens and alterations of several other genetic pathways. The age group in the present series is in accordance to many western studies showing laryngeal carcinoma presentation at later age^13^ may be related to cumulative molecular events increasing with time in relation to the duration of exposure and decreasing immune responses and general susceptibility.

The gender distribution in our study displayed very close male to female ratio, though the number of males in comparison to females is higher in most of the studies.^14^ We hypothesize that no remarkable difference for gender was noted, due to referral bias and larger number of HPV positive cases reported in females relate to the strong association of high risk HPV oncogenesis.

In our study the prevalence rate of HPV in laryngeal squamous cell carcinoma was found in 28 cases out of a total of 88 cases (31.8%), which was in concordance with several studies conducted^15^ showing prevalence of 26.02% and predominance of HPV genotype 16 whereas another study from Bangladesh^16^ showed HPV prevalence of 21% with genotypes 16 as the most common type. A local study showed 11% HPV positivity with prevalence of HPV 16 genotype.^17^ An Iranian study showed HPV prevalence of 25% with predominant genotype 16 & 18.^18^ This indicates the role of HPV in laryngeal carcinogenesis as a definitive possibility.

However, some of the studies differ with either very low or high HPV positivity for the varied sensitivity of different detection techniques or faulty tissue processing. Genetic diversity, environmental conditions, lifestyle and HPV detection method mostly explain the differential HPV infection rates in laryngeal squamous cell carcinoma in different regions. Another striking association of HPV with p16 immuno-expression is noted in our study in concordance to P16 being a surrogate marker. This is extensively reported in HPV positive cases of cancer from various sites.^19^

The significant expression of all molecular events pertaining to expression of Cyclin D1, p53, p16 and EGFR is displaying association with the progression of tumor Grade. This supports the role of cumulative molecular events to the progression of aggressive tumor phenotypes. Though we noted the significant correlation of HPV 16/18 with p16 and EGFR only. Mutation of Cyclin D1 was the most common molecular event in our series which supports the role of alternative pathways possibly in relation to tobacco related chemicals and other carcinogens. Several other studies have similarly reported the expression of oncogenes and anti-oncogenes in laryngeal carcinogenesis including the onco-proteins that we investigated.^20^-^21^

A significant finding in our study was that HPV positive laryngeal squamous cell carcinomas showed a high percentage of p16 expression which is proven to show better prognosis in LSCC. p16 is also known as a cyclin dependent kinase inhibitor 2A (CDKN2A), is a protein that plays a crucial role in regulating cell cycle and preventing uncontrolled cell growth. In HPV related cancers, the viral onco-proteins E6 and E7 bind and inactivate the tumor suppressor proteins p53 and pRb respectively leading to the dysregulation of cell cycle and promotion of cell proliferation.

It is postulated that the viral proteins and cell cycle regulators must be augmenting the molecular mechanisms of carcinogenesis in concert. The elucidation of these molecular events gives the targets and checkpoints for the development of consistent tumor markers and therapeutic interventions.

### Limitations:

Our limitation was the categorization of few unidentified high risk HPV genotypes and correlation with the clinical staging due to incomplete clinical information in certain cases. Moreover, stratification was possible in different grades as consecutive biopsies were included which are infrequent specimens for histopathology.

## CONCLUSIONS

The role of high-risk HPV 16/18 is concurred in the present study strongly in correlation to p16 as a surrogate marker. Moreover, the other driver mutations of Cyclin D1, p53, and EGFR are also implicated as cumulative molecular events in tumor progression as mostly seen in higher Grades.

### Future Recommendations:

We recommend further analysis of high risk HPVs except 16/18 to study the role of HPV in Laryngeal carcinogenesis. Moreover, the protein expression mutation profile can be validated by molecular genetics studies like Next Generation Sequencing (NGS) and microarrays to confirm the mutation profile in laryngeal oncogenesis with better accuracy in a larger number of cases.

### Author’s Contribution:

**NT:** Conception of the idea with bench work and manuscript writing. **TM:** Supervision of Histological and immunohistochemical procedures and review of manuscript. **MA:** Supervision of molecular genetics technique and editing of manuscript. **SN:** Technical assistance and bench work for molecular genetic techniques (PCR).
